# Effects of mind–body therapies on depression among adolescents: a systematic review and network meta-analysis

**DOI:** 10.3389/fpubh.2024.1431062

**Published:** 2024-07-10

**Authors:** Shulai Luo, Zhengyang Mei, Guisong Fang, Guogang Mu, Xiuying Zhang, Shi Luo

**Affiliations:** School of Physical Education, Southwest University, Chongqing, China

**Keywords:** mind–body therapies, adolescents, depression, network meta-analysis, yoga, dance therapy, Tai Chi

## Abstract

**Background:**

Depression poses significant threats to adolescents’ health globally. Research has shown the potential of mind–body therapies to alleviate depression, but limited studies have directly compared the therapeutic effects of different types of mind–body therapies on adolescent depression and the optimal therapy remain unclear. Therefore, we conducted a systematic review and network meta-analysis of randomized controlled trials that met the inclusion criteria to explore the effectiveness of different types of mind–body therapies as interventions to improve depression among adolescents, and to identify the most effective interventions.

**Methods:**

A comprehensive search of databases including PubMed, EMBASE, Cochrane Library, Web of Science, and Scopus up to January 2024 was conducted to assess the impact of mind–body therapies on depression among adolescents. The risk of bias of the included studies was evaluated using Cochrane Review Manager 5.4. STATA 18.0 was used for network meta-analysis. The node-splitting method was used to test the local inconsistency of the network meta-analysis. Funnel plots and the Egger’s test were utilized to assess the potential impact of bias in this study.

**Result:**

This network meta-analysis included 9 randomized controlled trials involving a total of 955 subjects. The results indicated that yoga, dance therapy and Tai Chi were more effective than other mind–body therapies in reducing symptoms of depression among adolescents. Specifically, according to the SUCRA ranking, yoga was rated to be the optimal intervention for adolescents with depression (SCURA: 82.2%), followed by dance therapy (SCURA: 77.5%) and Tai Chi (SCURA: 64.9%).

**Conclusion:**

This study revealed that mind–body therapies have positive effects on improving depression among adolescents. Yoga may be the most effective intervention among the different types of mind–body therapies. However, due to the small sample size of patients included, the certainty of the results was limited to some extent. Therefore, further investigation is necessary to strengthen the evidence base when more relevant studies become available.

**Systematic review registration:**

https://www.crd.york.ac.uk/PROSPERO/, identifier CRD42024508774.

## 1 Introduction

An estimated 3.8% of the population worldwide experience depression (1). Previous studies indicate that approximately 34% of adolescents worldwide are at risk of developing clinical depression, with the highest rates observed among adolescents living in the Middle East, Africa, and Asia (2). According to Report on National Mental Health Development in China (2021–2022), 24.1% adolescents were depressed, meaning that about one in four adolescents suffers from depression (3). Depression is a common, debilitating, burdensome, and chronic mental health problem that is prevalent during adolescence (4, 5). Stressors among adolescents, usually aged 10 to 19 (6), include physical and mental changes, academic pressure, and shifts in family and social relationships. At this time, due to the immature emotion regulation abilities of adolescents, long-term exposure to these stressors causes difficulty in coping with negative emotions, leading to malfunction of reward system (i.e., abnormal secretion of endorphins, dopamine, oxytocin, etc.) or the abnormalities in the hypothalamic–pituitary–adrenal (HPA) axis, which may provide the environment for the emergence of depression (7, 8). This mood disorder is broadly characterized by persistent feelings of sadness, loss of interest in activities, and impairment of daily functioning (1, 9). The damaging effects of depression also extend to adolescents’ social relationships to academic performance, and depression-induced suicide has become the second leading cause of death among adolescents (10–12). Moreover, adolescent depression has high comorbidity with other mental disorders such as anxiety, substance abuse, and conduct disorders, and is often associated with risky behaviors. Early onset of depression during adolescence presents a more severe depression in adulthood (i.e., longer episodes, higher recurrence rates, and more residual symptoms), and adverse psychosocial outcomes such as lower subsequent educational attainments, lower perceived social support (13–16). Rather than conceptualizing depression as qualitatively distinct “entities” (i.e., major depressive disorder, persistent depressive disorder, premenstrual dysphoric disorder, etc.) (9), current research proposed the dimensional model, which conceptualizes depression as a continuously process starting from subthreshold depression (17), with all individuals falling somewhere on the depressive symptom spectrum, distress and impairment occurring at the extremes of the continuum where the individual lies (4, 18–20). Since dimensional models have been proven to be more valid than traditional categorical models in examining the etiology and treatment of adolescent depression (19), we will also use the term “depression” in the rest of this review to broadly refer to various collections of depressive disorders and symptoms.

Medication and psychotherapy are the primary clinical interventions for depression among adolescents. However, despite the fact that these therapeutic approaches have been adequately implemented, they can only reduce the disease burden of depression by 30% (21). A significant number of patients received insufficient relief of their symptoms, while 50% suffered at least one recurrence of a depressive episode after 6–12 months of treatment (22). Previous studies denoted that antidepressant medication carried potential side effects and might increase the risk of suicidality among adolescents (23–25). For instance, Selective Serotonin Reuptake Inhibitors (SSRIs) in clinical trials have increased the intensity of suicidal predictors in depressed patients, such as dysphoria, anxiety, impulsiveness, aggressivity, agitation, etc. (26). Besides, although psychotherapy can be effective, many patients may refuse it due to the stigma related to their symptoms, and for some adolescent patients, especially in low-and middle-income countries, it is very expensive and unaffordable (1, 27). In this context, there is a paramount necessity to find cost-effective, side-effect-free, and easily accessible forms of treatment for depression in addition to options that already exist (28).

Due to its low cost, simplicity of implementation, varied forms and enjoyable nature, exercise therapy has become a prevalent adjunctive treatment for adolescent depression in recent years (29, 30). Research has demonstrated that the physiological mechanisms underlying the positive effects of exercise on depression are related to cytokines, monoamine neurotransmitters, inflammatory factors, neural systems and other factors in the body (31). However, mind–body therapies (MBTs), combining various forms of exercise, have been developed as an effective intervention for the treatment of depression by strengthening the mind’s capacity to interact dynamically with the body’s functions and symptoms, and to establish a strong connection between the brain, mind, body and behavior, in order to achieve overall health (32). To date, MBTs may include, but are not limited to, yoga, mindfulness-based meditation practices, Tai Chi, dance therapy, Qigong, and Buduanjin etc., and effects varied with different forms of exercise. For instance, yoga reducing stress and psychiatric symptoms, can be an effective treatment option for depression (33, 34). As it helps regulate negative emotions and attention, mindfulness training is essential for both preventing and treating depression (35). Traditional Chinese MBTs like Tai Chi, Qigong, and Buduanjin were proven to enhance patients’ neurocognition through mild movements combined with breathing relaxation techniques (36, 37). However, despite the growing variety of mind–body therapies from the East and West (32), there is a lack of evidence to support the efficacy of different MBTs for depression among adolescents, and the best non-pharmacological treatment strategy for depression in adolescents is unclear (38).

Therefore, the aim of this study is to assess the effects of different types of MBTs on depression among adolescents, and use a network meta-analysis to comprehensively compare and rank multiple different mind–body interventions, thus providing a basis for selecting the optimal treatment plan and informing clinicians when developing non-pharmacological treatment strategies.

## 2 Materials and methods

We have registered (CRD42024508774) this systematic review and network meta-analysis in the International Prospective Register of Systematic Reviews (PROSPERO), and followed the Preferred Reporting Items for Systematic Reviews and Meta-Analyses Network Meta-Analysis (PRISMA -NMA) statement for reporting. Ethical approval is not required as it is a systematic review.

### 2.1 Search strategy

We performed a systematic search following in the five electronic databases (PubMed, Embase, Cochrane Library, Web of Science, and Scopus) and used a snowball strategy to find relevant articles from their references and subsequent citations. The search was limited to English language literature and the period covered from the inception of each database until January 2024, and the search strategy followed the PICOS principle:

(P) population: adolescent patients with depression or depressive symptoms;

(I) intervention: mind–body therapies, including Yoga, Mindfulness, Tai Chi, Dance, etc.;

(C) control group: usual care including daily care, waitlist control conditions, routine exercise or other social activities;

(O) outcomes: The outcome measures included at least one of the following: the Patient Health Questionnaire (PHQ-9), the Beck Depression Inventory (BDI), the Symptom Checklist (SCL), the Center for Epidemiologic Studies Depression Scale (CES-D), and the Hamilton Rating Scale for Depression (HAM-D);

(S) study type: randomized controlled trials (RCTs).

The search strategy is provided in the Supplementary material, as the PubMed interface.

### 2.2 Exclusion criteria

The current study excluded: (1) non- randomized controlled trials, such as quasi-randomized controlled trials, animal studies, protocols, conference abstracts, case reports, etc.; (2) studies with incomplete or unreported data; (3) purely descriptive studies.

### 2.3 Literature screening

We utilized Endnote X9 literature management software to detect and exclude the duplications. Subsequently, two authors (SLL and ZYM) independently evaluated the titles and abstracts of the retrieved articles to ensure their eligibility for inclusion in the study. No further review was conducted for studies that did not meet inclusion criteria. Then, the two authors reviewed the full texts of the remaining literature. During this process, any disagreements were discussed to reach a resolution, or addressed by consulting another author (SL).

### 2.4 Data extraction and quality assessment

Three authors (GSF, GGM, and XYZ) independently extracted the data from selected RCTs including (1) basic information such as the first author’s name, year of publication, and country; (2) characteristics of the subjects, including mean age and gender; (3) experimental settings, including sample size, exercise type, cycle, frequency, and time; (4) primary outcome measures and measurement tools.

Risk of bias (ROB) assessment was conducted using Cochrane Review Manager 5.4 version, the assessment criteria included the following seven aspects: (1) Generation of the random sequence, (2) Hidden treatment allocation, (3) Blinding of study subjects or intervention personnel, (4) Blinding of result assessors, (5) Completeness of data results, (6) Selective reporting of results, and (7) Other bias. Each item was evaluated using “Low risk of bias,” “High risk of bias” and “Unclear risk of bias.” Trials were categorized into three levels of ROB by the number of components for which high ROB potentially existed: high risk (five or more), moderate risk (three or four) and low risk (two or less).

### 2.5 Statistical analysis

We utilized Stata 18.0 to perform a network meta-analysis and followed the PRISMA-NMA instruction manual. This method is particularly effective for data processing in multi-arm trials, providing comprehensive comparisons of multiple interventions while the statistical power and precision of the estimates are well-maintained (39). Considering that the outcome measures were continuous variables and the assessment scales were different across included articles, we used the standard mean difference (SMD) and 95% confidence interval (CI) as the effect size measure of the summarized results, with *p* < 0.05 indicating statistical significance. Statistical tests were used for evaluating heterogeneity, when *p* > 0.10 and I^2^ < 50%, indicating low heterogeneity, a common-effect model was used; while *p* < 0.10 and I^2^ > 50% indicated high heterogeneity and a random-effects model was used (40). A global consistency test was conducted to assess potential inconsistency between direct and indirect evidence, and node splitting was employed to ascertain local consistency (41). If the analysis showed *p* > 0.05, indicating that the direct and indirect comparisons did not have a significant difference, the effect sizes of multiple treatment comparisons could be analyzed using a consistency model. If not, an inconsistency model was applied. When there were closed loops present in the comparative studies, a test for inconsistency in the loops was conducted, with a 95% CI containing 0 denoting no significant loop inconsistency.

By comparing the surface under the cumulative ranking curve (SUCRA), rankings of different MBTs were established. A higher SUCRA value implies a higher probability of ranking (42). Network funnel plots were generated and visually monitored using symmetry criteria to assess if a small-scale study could result in publication bias in the network meta-analysis. In summary, the above methods for investigating the geometry of the treatment network and potential biases contributed to a more valid and reliable network meta-analysis result.

## 3 Results

### 3.1 Literature selection

**Figure 1:**
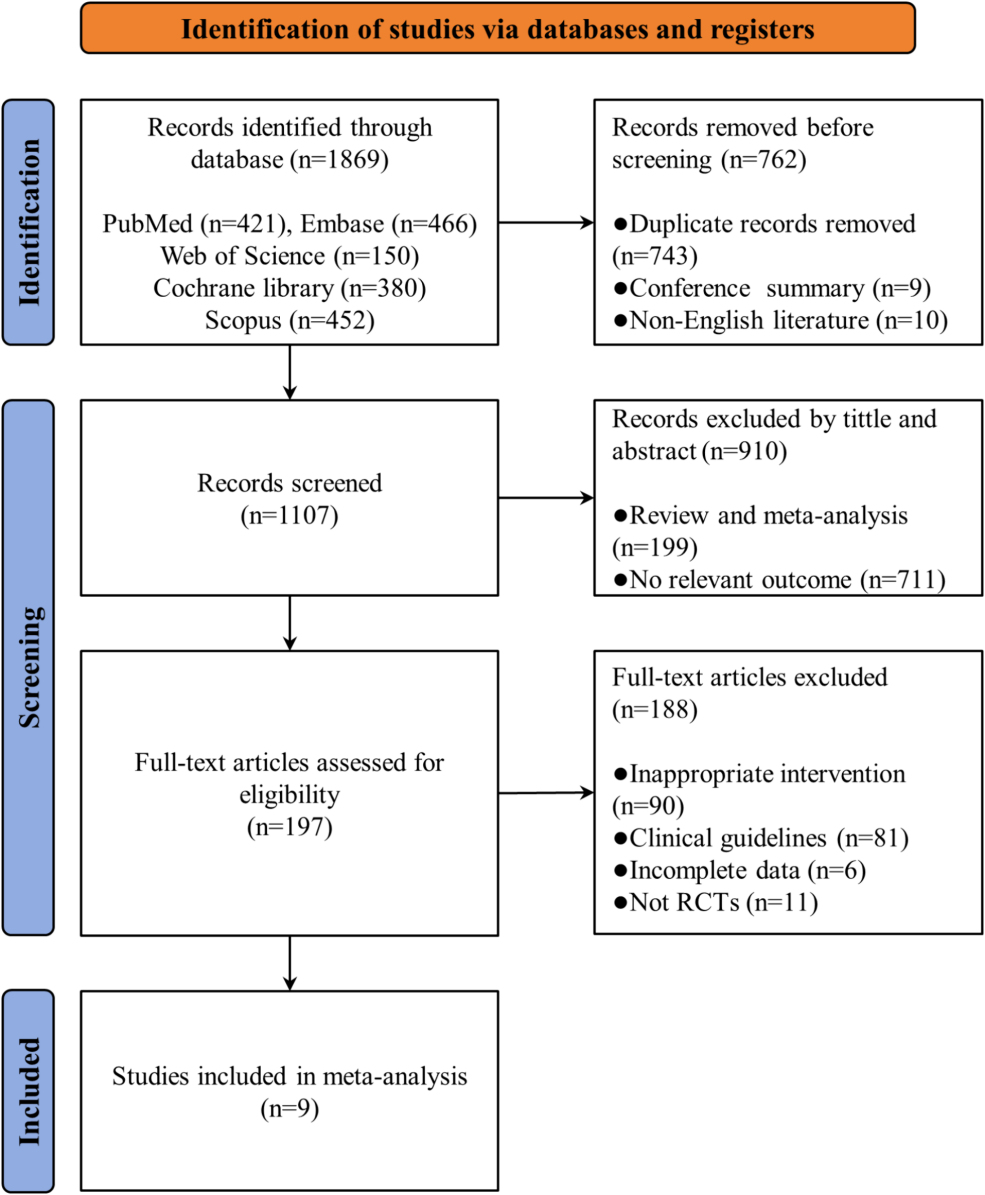
Flow diagram of literature selection.

A total of 1869 articles were identified from five databases aforementioned. After excluding 743 duplicates, 10 non-English literature and 9 conference abstracts, we completed a preliminary review of titles and abstracts, excluding 910 irrelevant articles and meta-analyses. The remaining 197 articles were eligible for the full-text review, after a comprehensive and careful review, 188 articles were excluded for reasons such as being non-randomized controlled trials, containing incomplete data, being improper article types like meeting abstract, or inappropriate intervention type. Finally, we included 9 published randomized controlled trials in this systematic review and network meta-analysis. The whole selection process has been illustrated in Figure 1.

### 3.2 Quality assessment of the included studies

**Figure 2:**
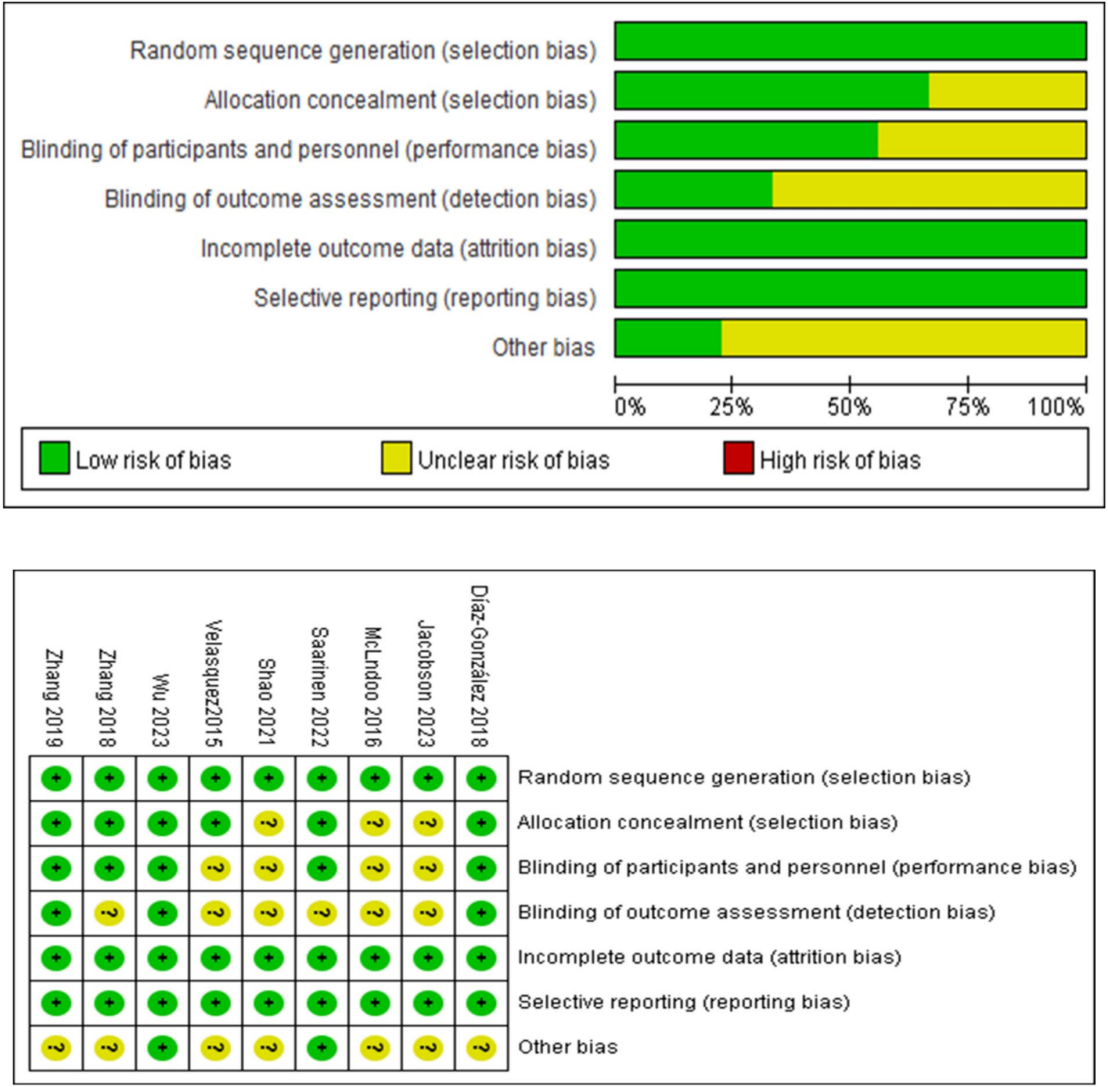
Quality assessment of included studies.

All 9 studies included in this review mentioned using methods like random number tables and computers to generate random sequences. Six studies allocation reported concealment methods such as opaque sealed envelopes. Five studies blinded patients, while only 3 blinded those who assessed the study results. The integrity of data in all 9 studies was generally good, and no signs of selective reporting were observed. Two studies showed no other biases, and for the remaining studies, other biases were unknown. The detailed quality assessment results are presented in Figure 2.

### 3.3 Basic characteristics of the selected studies

This study covers 9 randomized controlled trials, which involved a total of 955 adolescent patients with varying levels of depression. All participants were randomly assigned to the experimental group and control group. The experimental group was treated with a variety of interventions, including 4 involving mindfulness-based interventions, two involving yoga, two involving Tai Chi, and one involving dance therapy. Each intervention lasted 45 min–2 h, and the frequency varied from once a week to six times a week. The control group received interventions such as treatment as usual, physical exercises, and relaxation activities. The distribution of the included studies takes place in China (4), the United States (2), Spain (1), Finland (1), and Columbia (1), with the publication year mainly concentrated between 2015 and 2023. The outcome measurements varied across studies. Depression among adolescents were evaluated utilizing the Patient Health Questionnaire, Beck Depression Inventory, Revised Beck Depression Inventory, Symptom Checklist 90, and Strengths and Difficulties Questionnaire. The characteristics and details of the selected literature are summarized in Tables 1, 2.

**Table 1:** Basic characteristics of the included studies.

Study	Country	Age	Population	Total/male/female	Intervention	Control
Wu et al. (51)	China	T: 19 C: 19	Subthreshold depression	T:49/15/34 C:54/18/36	Tai Chi training Length of Intervention: 12 weeks; Freq: 3 times a week; Duration: 1 h.	Waiting list
Jacobson (44)	United States	T:NA C:NA	Depression of all levels (Including subthreshold)	T:30/NA/NA C:32/ NA/NA	Hatha yoga training Length of Intervention: 6 weeks; Freq: 3 times a week; Duration: 60–70 min.	Routine health program
Saarinen et al. (35)	Finland	T:NA C:NA C:NA	Subthreshold depression	T:166/57/109 C:157/47/110 C:46/9/37	Mindfulness-based Intervention Length of Intervention: 9 weeks; Freq: 5–6 times a week; Duration: 45 min.	Relaxation/No intervention
Shao (45)	China	T:NA C:NA	Subthreshold depression	T:32/15/17 C:30/15/15	Dance therapy Length of Intervention: 8 weeks; Freq: once a week; Duration: 2 h.	No intervention
Zhang et al. (46)	China	T: 19.1 C: 18.7	Subthreshold depression	T: 28/11/17 C: 28/13/15	Mindfulness-based stress reduction training Length of Intervention: 8 weeks; Freq: once a week; Duration: 1 h.	No intervention
Zhang et al. (47)	China	T:NA C:NA	Subthreshold depression	T: 32/NA/NA C:32/NA/NA	Mindfulness-based Tai Chi PE classes Length of Intervention: 8 weeks; Freq: Twice a week; Duration: 90 min.	No intervention
Díaz-González et al. (48)	Spain	T: 14.6: 14.5	Subthreshold depression	T: 41/18/23 C: 39/18/21	Mindfulness-based stress reduction training Length of Intervention: 8 weeks; Freq: once a week; Duration: 90 min	Treatment as usual
McIndoo et al. (49)	USA	T: 19.3 C: 19	Depression of all levels (Including subthreshold)	T: 20/8/12 C: 14/6/8	Mindfulness-Based intervention Length of Intervention: 4 weeks Freq: once a week; Duration: 1 h.	Waiting list
Velásquez et al. (50)	Colombia	T:NA C:NA	Subthreshold depression	T: 68/NA/NA C: 57/NA/NA	Yoga training Length of Intervention: 12 weeks; Freq: 2 times a week; Duration: 120 min.	Waiting list

**Table 2:** Details of participation controls for included studies.

Study	Participation inclusions	Participation exclusions
Wu et al. (51)	(1) Age between 18 and 25 years; (2) Score ≥ 16 on the Centre for Epidemiological Studies Depression Scale (CES-D); (3) No clinical intervention for depressive symptoms in the past 6 months; (4) Did not practice regular mind–body exercise in the past 6 months; (5) No contraindication to MRI examination.	The presence of suicide tendency as measured by the suicidality scale (cutoff score ≥ 6) of the Mini International Neuropsychiatric Interview (MINI version 5.0). The withdrawal criteria were: (1) volunteering to withdraw; (2) developing serious diseases that would not allow continuation of the intervention; (3) suffering from serious adverse events related to the intervention; (4) receiving Tai Chi training during the waitlist period.
Jacobson (44)	(1) The Beck Depression Inventory II, (BDI-II) scoring between 14 and 63; (2) Between the ages of 13–17; (3) Be able to do basic exercise. (4) Not to change medications if they were on them for depression.	(1) Having other debilitating mental disorders with the exception of anxiety; (2) Had history of suicide attempts.
Saeed et al. (35)	Not specified; invited comprehensive school students of appropriate age for participation and used a modified version of the Beck Depression Inventory (RBDI) to assess symptoms of depression.	Not specified.
Shao (45)	(1) The Symptom Checklist 90 (SCL-90) criterion of depression factor greater than 3.; (2) Those who are healthy and without physical diseases; (3) Those who volunteer to participate in group psychological counseling; (4) Those who are willing to complete the questionnaire survey carefully.	(1) Those who have serious psychological problems or mental diseases and are accepting drug treatment; (2) Those who are receiving psychological consultation or continuous systematic psychological counseling; (3) Those who are unwilling to complete the questionnaire survey; (4) Those with serious physical diseases.
Zhang et al. (46)	The Beck Depression Inventory (BDI) score > 14 and the Self-rating Depression Scale (SDS) score > 53	(1) Had recently suffered from major stress events; (2) Had major depressive disorder, bipolar disorder or other types of mental illnesses; (3) Had participated in or were participating in similar interventions (such as yoga or meditation)
Zhang et al. (47)	(1) Aged 16–19 years; (2) Had subthreshold symptoms of depression defined by nine-item Patient Health Questionnaire depression scale, (PHQ-9) as 2–4 symptoms of depression experienced more than half of the days or nearly every day for 2 or more weeks, which have affected study, social life, or functioning.	(1) Had other major disabling medical or mental disorder; (2) Had or being participated in a similar intervention; (3) Having major depression or no depression.
Díaz González et al. (48)	(1) Presence of psychological symptoms assessed by the Symptom Checklist 90 (SCL-90); (2) Age between 13 and 16 years.	(1) Having a neurological or psychiatric disorder that might interfere with participation (for example severe brain injuries, significant cognitive impairment, mental retardation, autism spectrum disorders, psychotic disorders, current suicidal ideation); (2) Current drug or alcohol misuse or dependence.
McIndoo et al. (49)	(1) The Beck Depression Inventory (BDI-II) score > 14; (2) Refrain from mindfulness practices, such as yoga, progressive muscle relaxation, and meditation during the study.	(1) Current alcohol and substance dependence and psychosis; (2) Medicated or not stabilized (i.e., same dosage for a minimum of 8 weeks) on antidepressant and antianxiety medication; (3) Receiving any current psychotherapy or counseling.
Velásquez et al. (50)	Not specified; selected appropriate aged students from public schools for participation and assessed symptoms of depression using the strengths and difficulties questionnaire (SDQ).	Not specified.

### 3.4 Network meta-analysis

**Figure 3:**
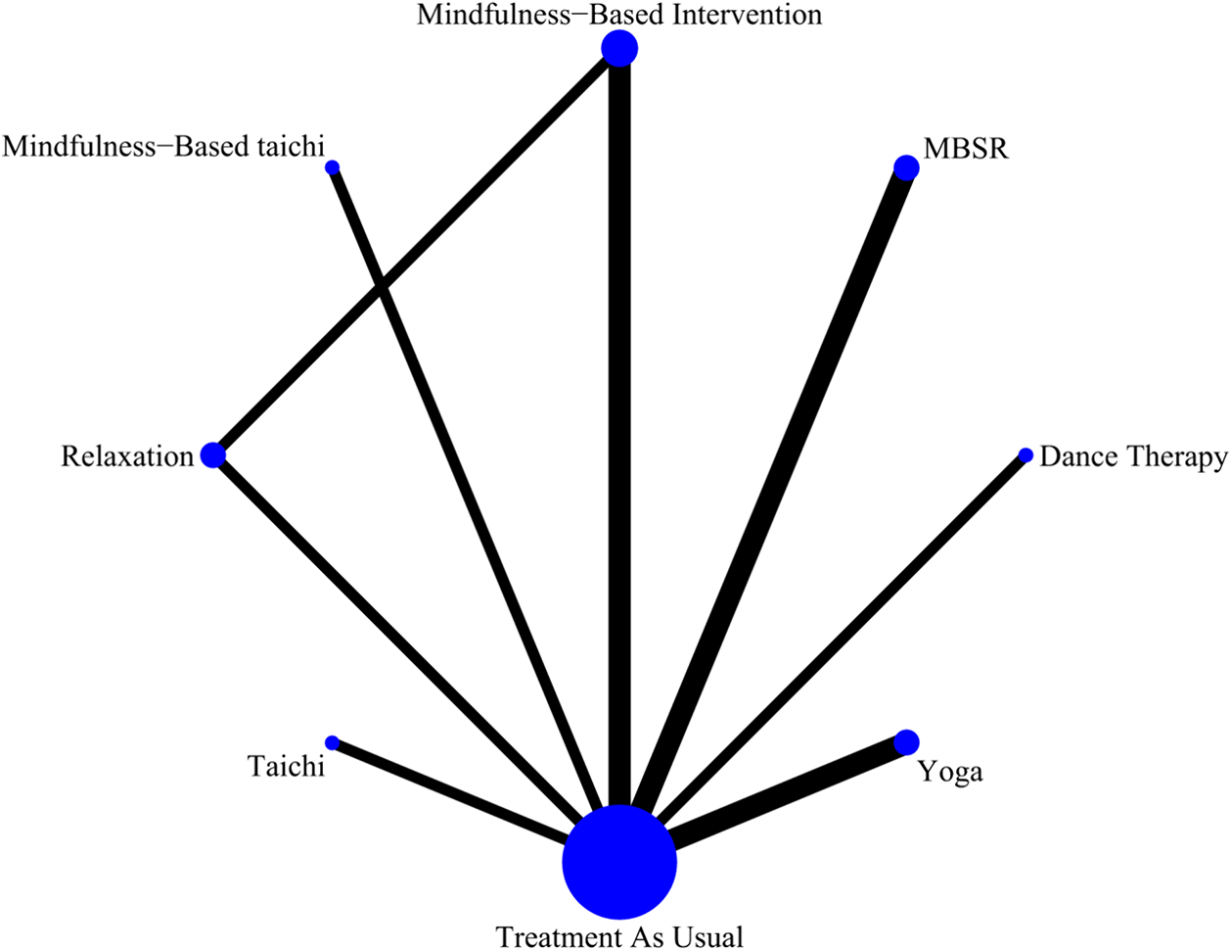
The network plot of the analyzed treatment comparisons for depression among adolescents.

**Figure 4:**
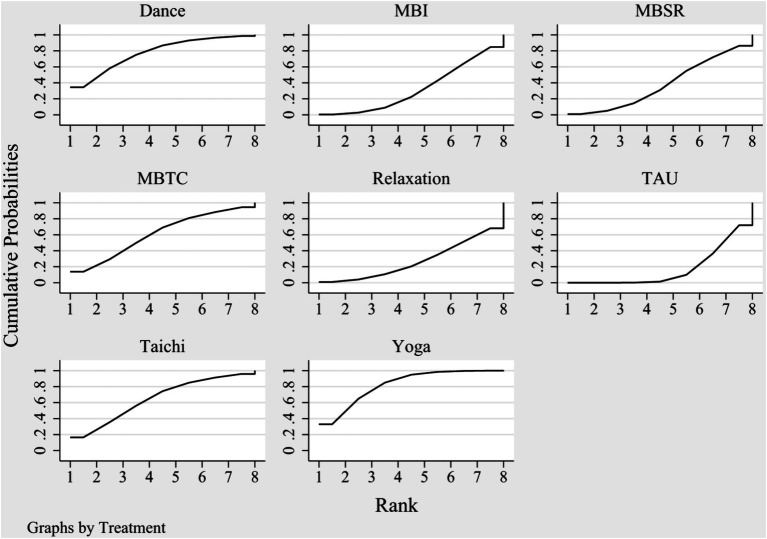
SUCRA score plot for depression among adolescents.

This study adheres to the principles of coherence, transferability, and consistency when conducting network meta-analysis. A network diagram (Figure 3) was established between different MBTs used to treat depression among participated adolescents. More specifically, the presence of the line between the two nodes in the diagram represents the direct comparison of studies, and vice versa. The thickness of the lines linking interventions reflects the number of studies comparing the two interventions. Figure 3 shows the comparisons among different interventions. Figure 4 demonstrates the rankings of interventions according to their potential to be the best choice for depression among adolescents (Table 3).

**Table 3:** League table for outcomes.

Yoga	−6.46 (−14.56, 1.63)	2.14 (−7.75, 12.03)	2.57 (−7.33, 12.48)	5.21 (−2.88, 13.30)	6.70 (−3.16, 16.57)	6.94 (−2.93, 16.80)	6.94 (1.18, 12.70)
6.46 (−1.63,14.56)	Dance	6.46 (−4.92,17.85)	6.23 (−5.16,17.61)	4.74 (−5.15,14.62)	−1.90 (−13.25,9.46)	2.10 (−9.32,13.52)	1.67 (−9.74,13.07)
−2.14 (−12.03,7.75)	−6.46 (−17.85,4.92)	Tai Chi	0.43 (−10.95,11.81)	3.07 (−6.77,12.91)	4.56 (−6.78,15.91)	4.80 (−6.55,16.14)	4.80 (−3.24,12.84)
−2.57 (−12.48,7.33)	−6.23 (−17.61,5.16)	−0.43 (−11.81,10.95)	MBTC	2.64 (−7.21,12.49)	4.13 (−7.23,15.49)	4.36 (−6.99,15.72)	4.37 (−3.69,12.42)
−5.21 (−13.30,2.88)	−4.74 (−14.62,5.15)	−3.07 (−12.91,6.77)	−2.64 (−12.49,7.21)	MBSR	1.49 (−8.32,11.30)	1.73 (−8.09,11.54)	1.73 (−3.94,7.40)
−6.70 (−16.57,3.16)	1.90 (−9.46,13.25)	−4.56 (−15.91,6.78)	−4.13 (−15.49,7.23)	−1.49 (−11.30,8.32)	MBI	0.24 (−7.77,8.24)	0.24 (−7.77,8.24)
−6.94 (−16.80,2.93)	−2.10 (−13.52,9.32)	−4.80 (−16.14,6.55)	−4.36 (−15.72,6.99)	−1.73 (−11.54,8.09)	−0.24 (−8.24,7.77)	Relaxation	0.00 (−8.01,8.01)
−6.94 (−12.70,-1.18)	−1.67 (−13.07,9.74)	−4.80 (−12.84,3.24)	−4.37 (−12.42,3.69)	−1.73 (−7.40,3.94)	−0.24 (−8.24,7.77)	−0.00 (−8.01,8.01)	TAU

The *p*-values for indirect and direct comparisons between studies were examined for global inconsistency. The effect of consistency between studies was acceptable since all *p*-values were above 0.05. The network relationship was centered on treatment as usual and formed three closed loops (Figure 3) which were subjected to local inconsistency tests using the node-splitting method, and the results indicated no significant inconsistency in the closed loops.

The results of network meta-analysis showed that yoga and dance therapy were significantly effective in reducing depression when compared to treatment as usual received by the control group. In the SUCRA ranking table (Figure 4), yoga was rated to be the optimal intervention for adolescents with depression with a probability of 82.2%, followed by dance therapy (77.5%) and Tai Chi (64.9%). According to the results of ranking, the effects of decreasing adolescents’ depression of different MBTs are as follow: yoga> dance therapy> Tai Chi> mindfulness-based Tai Chi> mindfulness-based stress reduction> mindfulness-based intervention> relaxation> control group.

### 3.5 Publication bias test

**Figure 5:**
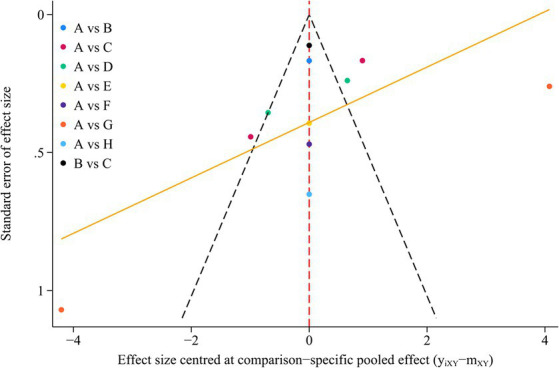
Funnel plot on publication bias.

We generated funnel plot for outcomes using STATA software version 18 to asses potential publication bias. The results showed that the symmetrical distribution of all the studies in the funnel plot could not be well observed, so we further conducted the Egger’s test for quantitative analysis of the publication bias test. The *p*-value in the Egger’s test was 0.09, indicating that there was no significant publication bias for the outcome indicators. The funnel plot is shown in Figure 5.

### 3.6 Sensitivity analysis

We performed sensitivity analyses by excluding individual studies, and the results showed no significant change in the statistical significance of the outcomes in this study. This further validates the robustness of our results.

## 4 Discussion

This network meta-analysis aimed to evaluate the effectiveness of different mind–body interventions on depression among adolescents and compared it with the control group. The results showed that MBTs such as yoga, dance therapy, Tai Chi, significantly alleviated depression levels compared to the control group interventions. These findings are consistent with the previous reviews with respect to the effects of MBTs addressing the mental health issues among adolescents (32). Therefore, the current review findings may provide referenceable evidence to apply MBTs to the young population. Pairwise comparisons revealed that yoga and dance therapy showed stronger effects on depression than other interventions. Based on the size of the efficacy and the ranking probability of the SUCRA values, yoga proved to be the most effective for depression among adolescents (SUCRA: 82.2%), followed by dance therapy (SUCRA: 77.5%) and Tai Chi (SUCRA: 64.9%).

Yoga is an ancient spiritual discipline that includes physical postures, movement control, breathing exercises, and mindfulness meditation (52–54). These methods facilitate the integration of the body, breath, thought and affect, thereby relieving stress and psychiatric symptoms (34, 55). Our findings revealed that yoga ranks high in SUCRA values for addressing depression in adolescents, demonstrating its potential as an optimal complementary treatment option. The pathogenesis of depression has not been elucidated clearly and is currently thought to be related to biological, genetic, environmental and psychosocial factors. Initially, it was thought that depression was associated with abnormalities in monoamine neurotransmitters, with dopamine being one of them (43). Dopamine is related to emotion regulation and plays an important role in the reward circuitry (56). By affecting the neurochemicals released in the brain, yoga is considered one of the most beneficial coping strategies for reducing stress (57). Many studies have proven the effects of yoga on the regulation of the dopamine system. During yoga meditation, there was a 7.9% reduction in ^11^C-raclopride binding in the ventral striatum, indicating a 65% increase in endogenous dopamine release (56). In addition, yoga induces complete relaxation of the body through simple to advanced breathing exercises that bring one into a meditative state, which can enhance inhibitory neuronal function (58–60), thereby helping to adjust the overreactive state of the dopamine system (61). However, recent theories suggest that depression is also related to more complex neuromodulator systems and neural circuits (43). Typical examples such as HPA axis dysfunction and inflammatory cytokines induced by psychosocial stressors may trigger depression (62–64). Specifically, yoga’s mindfulness techniques assist in stress reduction, exert a positive influence on an individual’s ability to self-regulate (65, 66), and inhibit the overactive HPA axis function (67). In addition, the yoga components of slow breathing, relaxation practices, mindfulness of sensations in the body can activate the vagal anti-inflammatory pathways (68, 69), which could be an essential mechanism whereby yoga practice reduces depression (70).

Dance therapy is the psychotherapeutic use of movements to promote emotional, social, cognitive, and physical integration in the individual, for the purpose of improving health and well-being (71). Over the decades, dance therapy has become a typical MBTs intervention prevailing in many regions of the world and across all age groups (72). Our results indicated that dance therapy ranked second only to yoga. The antidepressant effect of dance therapy can be explained from an endocrine perspective. Firstly, endorphins are chemicals naturally produced by the nervous system to control pain or stress which are commonly labeled as “feel-good” chemicals (73). The movement, music and rhythm involved in dance therapy can affect endorphin release by significantly altering mood, stimulating intense feeling states and even strengthening prefrontal cortex function (74–79). Moreover, oxytocin is a hypothalamic neuropeptide associated with interpersonal functioning, stress coping, social memories formation and prosocial behavior in humans (80–83). Research previously found negative correlation between severity of depression and serum oxytocin concentrations in a clinical population (84). Deficits in oxytocin levels make it difficult for depressed people to cushion the negative emotions of loneliness, which is a significant predictor of depression, via the perception of social support (85, 86). Dance therapy augments the mirror neuron system when using mirroring techniques (87, 88) to produce synchronized movements, which affects oxytocin (89), thus helping to alleviate depression. Furthermore, contrary to yoga and Tai Chi, dance therapy movements may be more vigorous and rhythmic, making it more appealing to adolescents, especially young girls. Thus, the social potential of this intervention for antidepressant impact cannot be ignored. Depressed adolescents who gather to practice dance and get in touch with others struggling with similar symptoms can help them overcome loneliness and promote a positive antidepressant effect (90).

Tai Chi is a traditional Chinese fitness practice well known for its slow, fluid movements, deep breathing and meditative elements. It is particularly favored by a large group of older people as being composed of a series of gentle movements. Many studies have investigated the effects of Tai Chi on the physical and mental health of older adults (91, 92). However, the high SUCRA rankings of Tai Chi in our findings support its applicability to the adolescent population. As mentioned previously, the difficulty in regulating negative emotions caused by various stressors is an important contributor to depression among adolescents (7). However, Tai Chi’s emphasis on meditation and the connection between mind and body promotes superb emotional management (93). Besides, the deep breathing practices of Tai Chi enable individuals to cope with stressors in a more composed manner by stabilizing the autonomic nervous system, creating full relaxation of the body and mind, decreasing tension and anxiety (94). Additionally, when practicing Tai Chi, the focus on fluid body postures and combined with meditation can enhance concentration and self-harmony as well as reduce negative thoughts, which is a key factor in alleviating anxiety and depression and enables individuals to better cope with depressive emotions (37, 95).

Overall, this study bridges the research gap involving the adolescent population. The results of our network meta-analysis suggest that MBTs, particularly yoga, possess potential value in the treatment of depression among adolescents. However, it is worth noting that antidepressant medications remain the first-line therapies for the treatment of depression. Therefore, what needs to be clarified is that our findings should not be viewed as evidence that over-emphasizes such supplementary therapies. We would like to call for future research to devote more efforts to combining non-pharmacological treatments such as mind–body therapies with traditional medication and psychotherapeutic interventions, and to explore their interactions and strengths, so as to make up for the shortcomings of the existing treatment system.

## 5 Limitations and future directions

There are several limitations in this network meta-analysis. Firstly, we focused only on the overall effects of MBTs due to methodological limitations of the study without considering the influence of other exercise factors such as frequency, duration and intensity of exercise. Second, few studies have reported on the treatment of depression among adolescents after a longer follow-up period (no less than 6 months), even though one study showed that the intervention was still effective in reducing depression levels after 6 months, we do not suggest that it provides sufficient evidence of the long-term effectiveness for adolescent depression. Moreover, although the adoption of MBTs for the adolescent population is an emerging and promising practice, some MBTs remain underutilized, such as Qigong, Buduanjin, and Pilates, whose effects on adolescent depression have not been explored in depth. Consequently, the types of mind–body therapies covered in our current study are limited. Lastly, due to the limited number of eligible studies, we included a small sample size. The included studies were of relatively low quality and relied on self-reported outcomes measured by scales. There were a few studies that kept some depressed patients on stable medication for the duration of the trial to ensure stability due to patient health concerns, as well as a small number of studies that did not explicitly report participation exclusions, so there was a possibility that the effects of MBTs could have been slightly interfered with. Therefore, the results should be construed cautiously and might be reconsidered by researchers in the future when more quality studies are available.

In forthcoming research endeavors, it would be beneficial to discuss and investigate the dose effects related to MBTs, including the optimal frequency, duration, and intensity. Moreover, the long-term effects of MBTs on depression among adolescents deserve further investigation. Finally, it is absolutely imperative that more rigorous, standardized, high-quality randomized controlled trials be conducted to validate and enhance the reliability of the current findings. In particular, where feasible, a broader range of mind–body interventions, including but not limited to Qigong, Buduanjin, Pilates, etc., should be used to provide a more scientific and comprehensive exploration of the clinical outcomes of MBTs on adolescent depression.

## 6 Conclusion

Previous studies may have overlooked the application of MBTs in the adolescent population. This study is the first to explore the effectiveness of different types of MBTs for the treatment of depression among adolescents. Our findings revealed that yoga, dance therapy, and Tai Chi were effective in reducing depression level, with yoga producing the greatest effect. Considering the challenges of implementing effective interventions and the economic burden of treating adolescent depression, our findings are of significant value. However, the certainty of the evidence is limited by the small sample size of patients included and the low quality of the studies, and further investigation is necessary to strengthen the evidence base when more relevant studies become available.

## Data availability statement

The original contributions presented in the study are included in the article/Supplementary material, further inquiries can be directed to the corresponding author/s.

## Author contributions

SUL: Writing – original draft, Writing – review & editing, Conceptualization, Data curation, Formal analysis, Funding acquisition, Investigation, Methodology, Project administration, Resources, Software, Supervision, Validation, Visualization. ZM: Supervision, Validation, Visualization, Writing – review & editing. GF: Data curation, Writing – review & editing. GM: Data curation, Writing – review & editing. XZ: Data curation, Writing – review & editing. SIL: Data curation, Formal analysis, Funding acquisition, Methodology, Supervision, Validation, Writing – review & editing.
