# Supplementary material for: Effects of mind–body therapies on depression among adolescents: a systematic review and network meta-analysis
Source: Front Public Health. 2024 Jul 10;12:1431062. doi: 10.3389/fpubh.2024.1431062 (PMC11266190; doi:10.3389/fpubh.2024.1431062)
Supplement: Supplementary file 1 [file Data_Sheet_1.docx]

Supplementary Material

# Supplementary Figures and Tables

## Supplementary Figures


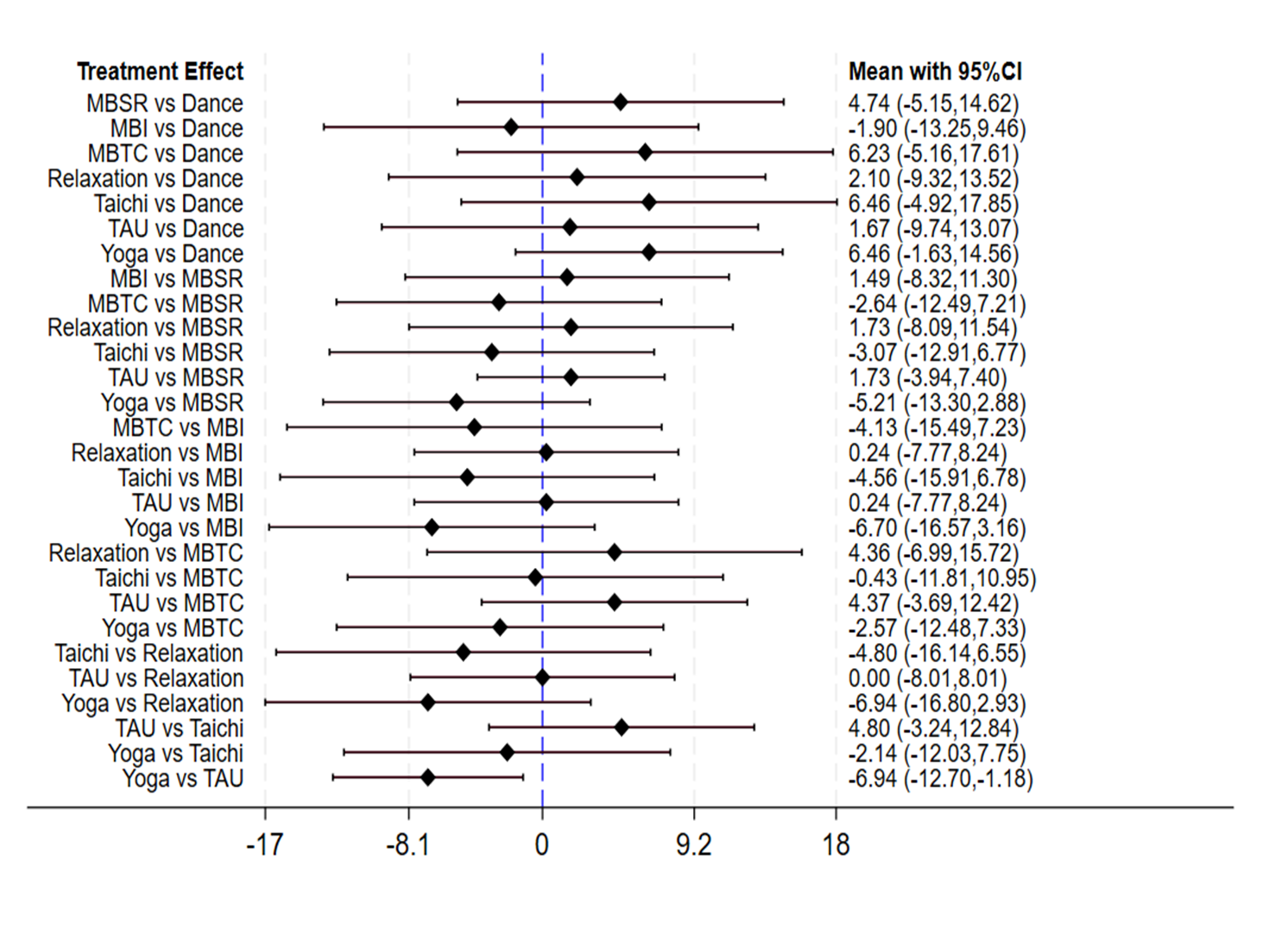


**Supplementary Figure 1.** Interval plot of network meta-analysis for depression among adolescents.

**
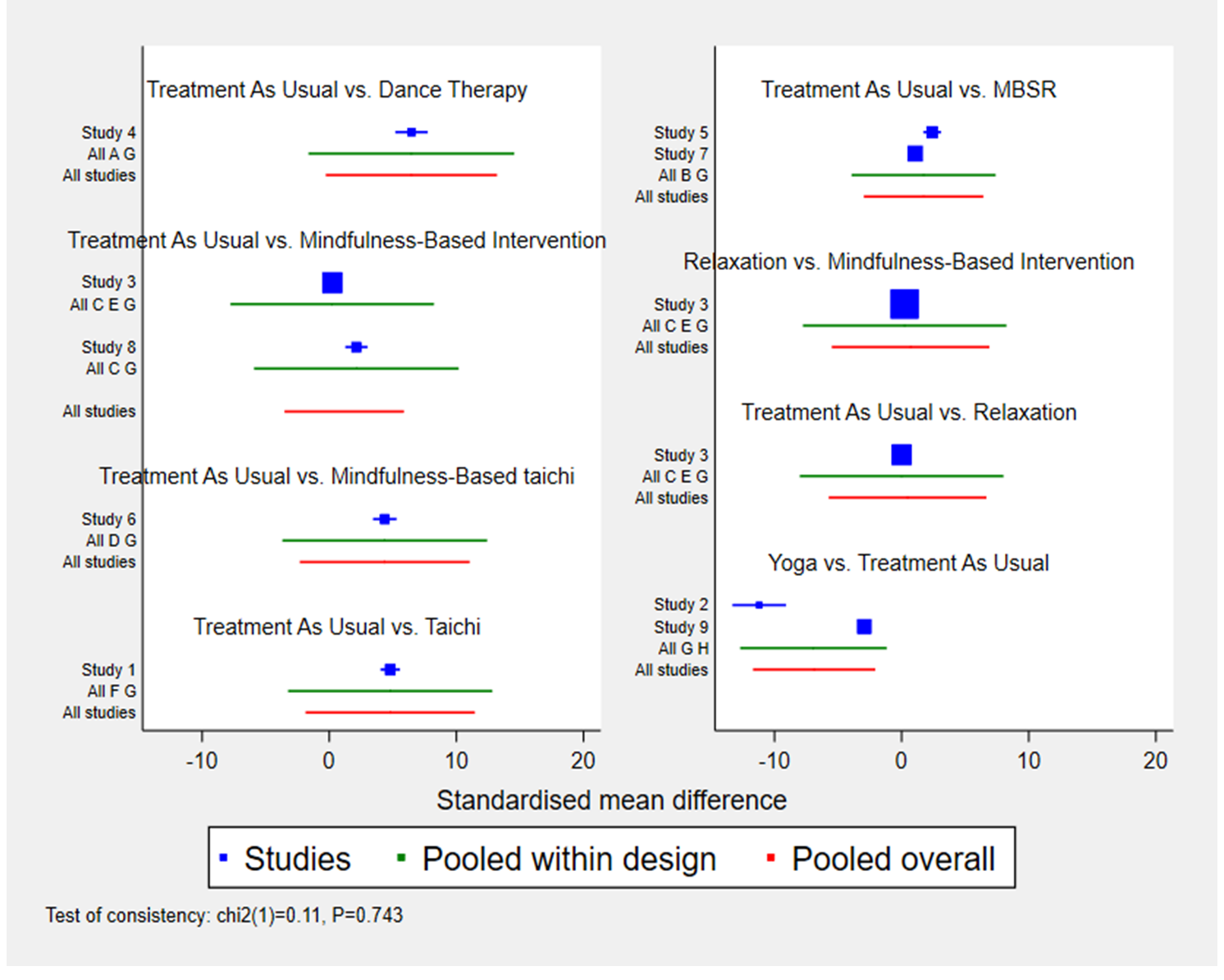
**

**Supplementary Figure 2.** Forest map of the effect of MBTs on adolescent depression.


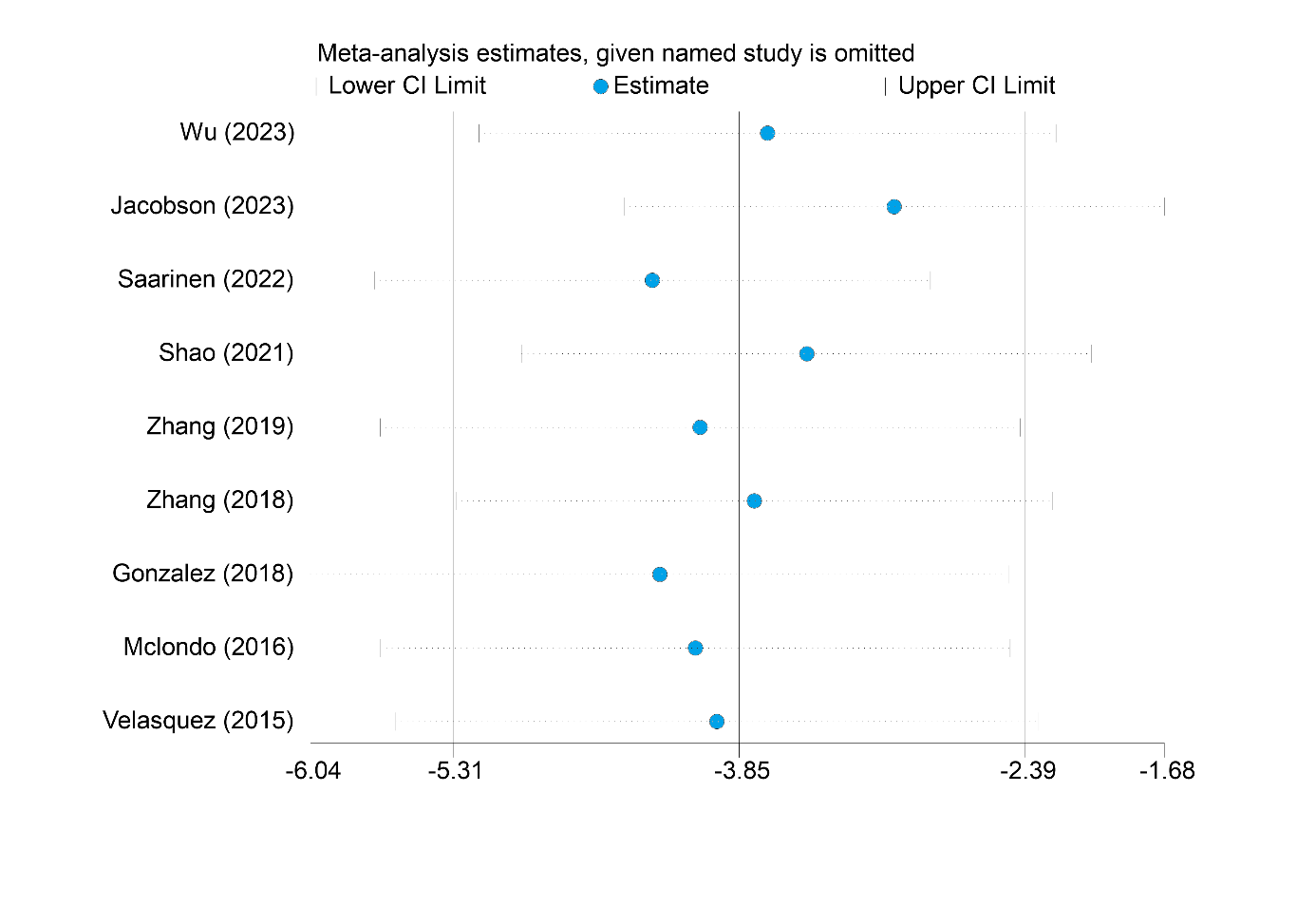


**Supplementary Figure 3.** Sensitivity analysis of depression among adolescents.

## Supplementary Tables

**Supplementary Table 1.** Search strategy in PubMed

| **Step** | **Search strategy** |
| --- | --- |
| #1 | (Depression [Mesh]) |
| #2 | ((((((Depressive disorder) OR (Depressive disorders)) OR (Depressive Symptoms)) OR (Depressive Symptom)) OR (Symptom, Depressive)) OR (Emotional Depression)) OR (Depression, Emotional) |
| #3 | #1 OR #2 |
| #4 | (((((((Mind-Body Therapies [MeSH Terms]) OR (mind body practice)) OR (Therapy, Mind-Body)) OR (Mind Body Therapies)) OR (Therapies, Mind-Body)) OR (Mind-Body Therapy)) OR (Mind-Body Medicine)) OR (Mind Body Medicine) |
| #5 | ((((((((((((((((((((((((((((((Tai Chi) OR (Qigong)) OR (Yoga)) OR (exercise movement techniques)) OR (Meditation)) OR (Mindfulness)) OR (Tai-ji)) OR (Chi, Tai)) OR (Tai Ji Quan)) OR (Ji Quan, Tai)) OR (Tai Ji)) OR (Taiji)) OR (Taijiquan)) OR (T'ai Chi)) OR (Tai Chi Chuan)) OR (Ch'i Kung)) OR (Movement Techniques, Exercise)) OR (Exercise Movement Technics)) OR (Pilates-Based Exercises)) OR (Exercises, Pilates-Based)) OR (Pilates Based Exercises)) OR (Pilates Training)) OR (Training, Pilates)) OR (Transcendental Meditation)) OR (Meditation, Transcendental)) OR (Mind Body Therapies)) OR (Mind-Body Therapy)) OR (Therapies, Mind-Body)) OR (Therapy, Mind-Body)) OR (Mind-Body Medicine)) OR (Mind Body Medicine) |
| #6 | #4 OR #5 |
| #7 | ((((((((((((((((Adolescent[MeSH Terms]) OR (Adolescents)) OR (Adolescence)) OR (Teens)) OR (Teen)) OR (Teenagers)) OR (Teenager)) OR (Youth)) OR (Youths)) OR (Adolescents, Female)) OR (Adolescent, Female)) OR (Female Adolescent)) OR (Female Adolescents)) OR (Adolescents, Male)) OR (Adolescent, Male)) OR (Male Adolescent)) OR (Male Adolescents) |
| #8 | (((Randomized Controlled Trials as Topic[MeSH]) OR (Randomized Controlled Trial[Publication Type])) OR (randomized[Title/Abstract])) OR (placebo[Title/Abstract]) |
| #9 | #3 AND #6 AND #7 AND #8 |

**Supplementary Table 2.** Risk of bias assessment

| Study | Random sequence generation | Allocation concealment | Blinding of participants and personnel | Blinding of outcome assessors | Incomplete outcome data | Selective reporting | Other biases |
| --- | --- | --- | --- | --- | --- | --- | --- |
| Wu 2023 | low | low | low | low | low | low | low |
| Jacobson 2023 | low | unclear | unclear | unclear | low | low | unclear |
| Saarinen 2022 | low | low | low | unclear | low | low | low |
| Shao 2021 | low | unclear | unclear | unclear | low | low | unclear |
| Zhang 2019 | low | low | low | low | low | low | unclear |
| Zhang 2018 | low | low | low | unclear | low | low | unclear |
| Díaz-González 2018 | low | low | low | low | low | low | unclear |
| McIndoo 2016 | low | unclear | unclear | unclear | low | low | unclear |
| Velásquez 2015 | low | low | unclear | unclear | low | low | unclear |
